# Dry eye disease in type 2 diabetes mellitus; comparison of the tear osmolarity test with other common diagnostic tests: a diagnostic accuracy study using STARD standard

**DOI:** 10.1186/s40200-015-0157-y

**Published:** 2015-04-29

**Authors:** Laily Najafi, Mojtaba Malek, Ameneh Ebrahim Valojerdi, Mohammad E Khamseh, Hossein Aghaei

**Affiliations:** Endocrine Research Center, Institute of Endocrinology and Metabolism, Iran University of Medical Sciences (IUMS), Firouzgar alley, Valadi St., Behafarin St., Karimkhan Ave., Vali- Asr Sq., Tehran, 15937-48711 Iran; Eye Research center, Rassoul Akram Hospital, Iran University of Medical Sciences, Tehran, Iran

**Keywords:** Diabetes mellitus, Dry eye disease, Diagnostic accuracy Study, STARD

## Abstract

**Background:**

To determine the diagnostic performance of tear osmolarity in diagnosis of dry eye disease by using tear lab osmolarity system in people with type 2 diabetes, and to compare it with common diagnostic tests already available in clinical practice.

**Methods:**

Two hundreds forty three people with type 2 diabetes were included. Tear osmolarity was measured with the tear osmolarity system. The 308 mOsm/L cutoff was used to diagnose dry eye disease. The following tests were also performed: Ocular Surface Disease Index (OSDI) questionnaire, Tear Film Break up Time (TFBUT), Schirmer I test, Rose Bengal and Fluorescein staining. The results of these tests were compared to the tear osmolarity measurement.

**Results:**

The prevalence of dry eye disease detected by the tear osmolarity test was 27.7%. It was as follows for the other common diagnostic tests: OSDI (17.7%), Schirmer I test (33%), TFBUT (41%), Rose Bengal (11%), and Fluorescein staining (4%). Fluorescein staining had the highest specificity (97%). With the cutoff score >12, the positive likelihood ratio for the OSDI questionnaire was the highest (1.78). The sensitivity was poor for all common diagnostic tests. ROC curve analysis could not determine optimal cut offs for the common diagnostic tests.

**Conclusions:**

The available common diagnostic tests underestimate the presence of dry eye disease in people with type 2 diabetes. Moreover, they could not discriminate tear hyperosmolarity from normal. Tear osmolarity could be considered as the best single test for detection of dry eye disease in people with type2 diabetes.

## Introduction

In adult population, dry eye disease (DED) is a common ocular disease and has been recognized as an important public health problem in recent years [1-3].

Although our knowledge about its pathogenesis, classification, and characteristics has grown considerably over the past decade, there are still debates on diagnostic approach. This is mainly due to the lack of objective tests with sufficient sensitivity and specificity that could be applied easily in routine clinical care settings and at the same time be adequately reproducible [4,5].

Schirmer I test, tear film break-up time (TFBUT) analysis, Rose Bengal and fluorescein staining are the most well-known objective tests used for diagnosis of DED [6]. However, neither symptoms nor signs always match with the results of these tests [7].

Tear osmolarity measurement by freezing point depression technique has been proposed as the gold standard test for diagnosis of DED [8], however, the problems associated with the existing technologies hindered its use in clinical practice [9]. Moreover, it is costly, time-consuming; and requires tear volumes much higher than those collectable in several forms of dry eye disease, or inducing excessive reflex tearing during tear sampling. Osmolarity microchip system appears to overcome these barriers. It measures tear osmolarity based on electrical impedance technique and the results correlate well with the gold standard freezing point depression technique [9].

Several previous studies, investigated the relationship between diabetes and DED [10]. It has shown that composition of tear proteins in people with diabetes is different from healthy subjects [11]. In diabetes mellitus corneal and conjunctival epithelial alterations, persistent epithelial defects, and potential visual impairment due to corneal scaring have been observed [12-18]. Damage to the microvasculature of the lacrimal glands accompanied with autonomic neuropathy could impair lacrimation in long standing diabetes [19].

The purpose of the present work was to determine the diagnostic performance of tear osmolarity test; used to diagnose DED in type 2 diabetes mellitus using tear lab osmolarity system as the reference standard and to compare it with the other diagnostic tests (index tests) already in use, specifically Ocular Surface Disease Index (OSDI) questionnaire, Schirmer I test, TFBUT, Rose Bengal and fluorescein staining.

## Methods

We prospectively enrolled 243 consecutive diabetic patients at the Institute of endocrinology and metabolism between August 2011 and November 2012.

Exclusion criteria include use of medications or history of any other ocular or systemic disease that can affect tear production or quality, history of anterior segment surgery, Keratorefractive procedures (LASIK, LASEK, PRK) within one year prior to enrollment, trauma, contact lens wear, incomplete lid closure, entropion, ectropion, nasolachrymal drainage obstruction, punctual plugs placement, or cauterization; ocular allergy, glaucoma, pregnancy or lactation, and use of ocular medications or nutritional tear supplements.

All of the participants underwent a general physical examination and a thorough ophthalmologic exam. The visual acuity of both eyes was tested using Snellen’s chart. Both eyes were examined first using the board beam of the slit lamp to know the condition of the ocular surface and adenexa, observing the tear film meniscus, tear film, corneal changes, conjunctival changes, and eyelids. They were also clinically evaluated with direct and indirect ophthalmoscopy to know the status of retina. The study population underwent tear osmolarity test (standard test) before the Ocular Surface Disease Index (OSDI) questionnaire, Schirmer I test, Rose Bengal and fluorescein staining, and tear film break-up time test (TFBUT) (index tests); all were performed on the same day. All of the tests were performed according to the randomization table for one eye.

OSDI questionnaire was administrated to the participants by a research associate trained by an expert ophthalmologist to score the questions and to follow the ambiguous information. Schirmer I test, Rose Bengal, fluorescein staining, TFBUT were performed for all of the participants by an expert ophthalmologist in consecutive sessions. Tear osmolarity test was done by a single specialist. The investigators were blinded to the patients’ history and the obtained information.

To avoid diagnostic error, all of the examinations were performed in the same physical condition and in the morning to standardize the tests and to avoid possible diurnal variation. Assessments were made in a room controlled for enlightment (dim light), temperature, humidity, and airflow, to avoid ocular surface stress. The tests that need slit lamp were performed in a darkened room with the same slit lamp and by the same physician.

Tear osmolarity was measured using tear lab osmolarity system, (BON Co. Germany). The Tear lab instrument is based on a lab- on – a- chip technology working as both a collection device and an analytical system, in absence of any chemical reagent. This avoids the need for a capillary tube or absorbing acetate disc. The equipment consists of single use test cards containing microchannels to collect tear fluid, held by a pen designed to facilitate tear collection, and a portable reader unit which elaborates and displays the osmolarity results. A tear sample, approximately 50 nl, was collected from the inferior lateral tear meniscus of the ocular surface. To facilitate tear collection, patients were asked to position their head laterally for a few seconds before approaching the tip of the test card microchip: in this way, tears were driven laterally and collection made easier. Subjects had been requested not to wear makeup on their eyelids. Quality control procedures were applied at the beginning of each day of patient testing by using reusable electronic check cards (provided by the manufacture as a procedural quality control) to confirm the function and calibration of the TearLab osmolarity system. The 308 mOsm/L cutoff was used to diagnose DED [20]. At this diagnostic cutoff, osmolarity was found to have 88% specificity, 75% sensitivity in mild/moderate disease and 95% sensitivity in severe disease [20].The very rapid acquisition of tear samples by the TearLab would be less likely to be influenced by evaporation.

The OSDI questionnaire has a Likert design and assesses frequency of ocular subjective symptoms (soreness, blurred vision), difficulty with vision-related function (television, visual display unit, driving, reading) and discomfort due to environmental triggers (low humidity, high wind). The patients answer 12 questions, with higher scores representing greater disability [21].

Subjective symptoms of dry eye were graded on the basis of dry eye discomfort symptoms questionnaire (OSDI) [9]. The score range was from 0–12 (no disability), 13–22 (light dry eye), to 23–32 (moderate dry eye), and 33–100 (sever dry eye) [9].

Schirmer I test and TFBUT were carried out as outlined in the DEWS report [4].

The Schirmer test is an invasive and indirect method to measure change in volume of the tears in the tear reservoir. This test involves insertion of a wick into the lower conjunctival sac and measurement of the wetting length over a set period of time. The Schirmer test uses filter papers to assess tear production. There are two commonly used variations of the Schirmer test: Schirmer I measures total tear secretion (reflex and basal tears) and Schirmer II utilizes anesthetic to measure basal secretions, although this has not been validated [22-24].

The strip was folded at the notch and placed at the junction of the middle and lateral thirds of the lower eyelids and allowed there to stay in place for 5 minutes [25].

A value of less than 5 mm wetting in 5 minutes is considered abnormal, more than 10 mm per 5′ seconds as normal and 6-10 mm per 5′seconds as borderline [4]. Medial and lateral placements of the paper have been described, as well as having the patient looking up, but no method has been deemed more reliable [26].

TFBUT is the standard test for estimating tear film stability. The results are explained as seconds. Patients with break-up time of more than ten seconds was consider as normal, those with less than ten seconds was labeled as unstable tear film, 6 to 10 seconds as moderate dry eye disease, and ≤5 sec as sever dry eye disease [9].

The TFBUT was performed by applying a fluorescein strip after moistening it with a drop of sterile saline, to the lower tarsal conjunctiva without the use of topical anesthesia. The time lapse between the last blink to the appearance of the first random dry spot was taken as the tear film break up time [25].

Epithelial damage to the exposed surface of the eye can be demonstrated with vital and supra-vital stains. Fluorescein and Rose Bengal staining are the standard but invasive methods used to demonstrate ocular surface damage. This technique reveals surface damage on both the cornea and conjunctiva [27].

The eye is best viewed with anexciter (Wratten 47/47a; Edmund Optics, Barrington, NJ, USA) and barrier filter (Wratten 12/15) to assess staining on the cornea and conjunctiva.

Evaluation of staining is highly subjective, but the use of charts such as the Oxford grading scheme can help by enabling consistent recording of staining severity and is used to estimate surface damage in dry eye. The scheme has five panels, labeled A--E, with staining represented by punctuate dots that increase logarithmically between the panels [28]. The clinician compares the appearance of staining on the exposed interpalpebral conjunctiva and cornea with each panel and the closest match determines the grade. On a specially designed form, a grade between 0 and 3was given for staining on the cornea, based on the number of “dots” seen, which was to be added to a grade between 0 and 3 for the nasal and the same for the temporal conjunctiva. This gave a maximum possible total of 9 points. Three additional points were then allocated for fluorescein only if there was confluent staining (+1), staining in the papillary area (+1), or one or more filaments (+1), giving a maximum possible score of 12. This was performed for each eye. An abnormal score was considered to be 3 and above [21].

The study was approved by the local ethical committee of Tehran University of Medical Sciences and conducted in accordance with the ethical principles of the Declaration of Helsinki and all of the participants signed the written informed consent.

### Statistical analysis

Statistical analysis was performed using Statistical Package for Social Sciences (SPSS, SPSS Inc., Chicago, IL, USA) version 18.0. Descriptive statistics were summarized as mean ± SD, or median and interquartile. Kolmogorov Smirnov was performed to assess normality for continuous variables.

An evaluation was made of the linear relationship between the tear osmolarity values and TFBUT, OSDI score, Schirmer I test and Rose Bengal and fluorescein staining. This was performed by using Spearman’s correlation coefficient (rho). Student’s *t* test and Mann–Whitney U test was applied for comparisons between two groups (significance *p* < 0.05).

Chi square test was used to compare discrete variables. Results were given with their 95% CIs.

Diagnostic accuracy tests was performed to analyze sensitivity, specificity, receiver operating characteristic (ROC) curves, positive likelihood ratio (LR+), and positive predictive values (PPV).

## Results

From August 2011 until November 2012, we prospectively enrolled 243 people with type 2 diabetes at Institute of endocrinology and metabolism. Four patients were excluded from the study for the following reasons: LASIK within the 1 year prior to enrollment (2), contact lens wear (1), and glaucoma (1).

The data from 137 female and 102 male were used for the final analysis. The mean age was 55.8 ± 10.33 years old, and the mean duration of diabetes was 9.08 ± 7.9 years. The mean for fasting blood glucose was 152.4 ± 59.6 mg/dl, and for HbA1C was 7.55% ± 1.73%. Oral glucose lowering drugs (OGLDs) were used by 69.4% of the participants, while 10.5% were on insulin only, and 15.5% were on OGLDs plus insulin.

Tear osmolarity measurements were successfully performed at the first attempt in all participants; no reflex tearing was observed in any subjects. Considering of dry eye disease definition; two groups were identified: the dry eye disease (DED) group (67), and the normal group (172). All of the analyses were done considering this classification.

Table 1 illustrates clinical characteristics of the participants, according to the tear osmolarity test categorization.

**Table 1:** **Clinical characteristics of the participants**

	**All participants**	**DED** ^**d**^ **group**	**Normal group**	**P-value**
		**(n = 67)**	**(n = 172)**	
**Age (yrs.)**				
**Mean ± SD**	55.8 ± 10.33	56.50 ± 9.47	55.43 ± 10.66	0.48
**Range**	22-84	37-81	22-84	
**Gender (female) n (%)**	137 (58%)	42(64%)	95(56%)	0.28
**BMI** ^**a**^ **(kg/m2)**	29.2 ± 4.9	29.42 ± 5.36	29.36 ± 5.12	0.94
**Diabetes duration (yrs.)**	9.08 ± 7.9	9.73 ± 8.25	8.71 ± 7.69	0.32
**FBS** ^**b**^ **(mg/dl)**	152.4 ± 59.6	163.63 ± 61.27	147.44 ± 59.61	0.09
**HbA1c** ^**c**^ **(%)**	7.55 ± 1.73	7.94 ± 1.87	7.14 ± 1.66	0.054

^a^BMI, Body Mass Index.

^b^FBS, Fasting blood sugar.

^c^HbA1C, Hemoglobin A1C.

^d^DED, Dry eye disease.

Data are mean ± SD unless otherwise indicated.

Statistical method: Mann–Whitney test, Independent sample t-test and χ2 –test.

The mean value for the tear osmolarity in all of the participants was 301.97 ± 13.52 mOsm/L. This value was 319.03 ± 7.92 mOsm/Lin the DED group and 295.38 ± 8.49 mOsm/Lin the normal group.

The prevalence of DED detected by the tear osmolarity test was 27.7% (10.2% in male, and 17.5% in female). The prevalence of DED detected by the other five diagnostic tests was as follows: OSDI (17.7%), Schirmer I test (33%), TFBUT (41%), Rose Bengal (11%), and fluorescein staining (4%).

Comparison of the results for all of the applied tests showed statistically non-significant difference between the two groups which is mentioned in Table 2.

**Table 2:** **Comparison of the OSDI, Schirmer I test, Rose Bengal staining, fluorescein staining, and TFBUT test results between the two groups**

	**All participants**	**DED group**	**Normal group**	**P-value**
		**(n = 67)**	**(n = 172)**	
**OSDI** ^**a**^ **(score)**	2.08 (0, 8.3)	2.08 (0, 14.58)	2.08 (0, 8.3)	0.44
**Fluorescein staining (score)**	0 (0, 0)	0 (0, 0)	0 (0, 0)	0.70
**Rose Bengal staining (score)**	0 (0, 1)	0 (0, 1.25 )	0 (0, 1)	0.13
**Schirmer I test (mm)**	5 (2, 10)	6 (2, 11)	5 (2, 10)	0.64
**TFBUT** ^**b**^ **(secs)**	8 (5, 12.25)	9 (5,13.25 )	8 (5,12)	0.27

^a^OSDI, Ocular Surface Disease Index.

^b^TFBUT, Tear film break-up time.

For all variables, data are shown as Median (Interquartile), Kolmogorov Smirnov normality test distribution.

Statistical method: Mann–Whitney test.

We also compared the discriminative ability of the OSDI, Schirmer I test, Rose Bengal and fluorescein staining, and TFBUT tests results in Table 2 to detect DED compared to the tear osmolarity test.

Considering adverse events, only three patients reported eye discomfort following conjugate staining for a few hours.

Due to technical failure, we could not interpret the test results for Schirmer I test and TFBUT in ten subjects. In addition performing ophthalmic tattoos was the reason for indeterminant test results in two subjects.

The correlation coefficient for the measured tear osmolarity and the results of the other diagnostic tests were calculated in Table 3. No significant correlation was found

**Table 3:** **DED detection rate by OSDI, Schirmer I test, Rose Bengal, fluorescein staining, and TFBUT compared to tear osmolarity test**

	**OSDI** ^**a**^	**Schirmer I test**	**Rose Bengal staining**	**Fluorescein staining**	**TFBUT** ^**b**^
Dry eye disease (n = 67)	14(20.8%)	20(29.8%)	8(11.9%)	3(4.4%)	27(40.2%)
Normal (n = 172)	21(12.2%)	48(27.9%)	14(8.1%)	5(2.9%)	57(33.1%)

^a^OSDI, Ocular Surface Disease Index.

^b^TFBUT, Tear film break-up time.

Data are n. (%).

.

The diagnostic performance for all of the diagnostic tests was determined. Data were summarized for sensitivity, specificity, positive predictive value (PPV), likelihood ratio (LR+), and area under the curve (AUC) from the ROC curve analysis in Table 4.

**Table 4:** **Correlation coefficient of the tear osmolarity and other diagnostic tests**

**Variable**	**Correlation coefficients (** ***r*** **)**	**P-value**
**Rose Bengal staining**	0.12	0.08
**OSDI** ^**a**^	0.11	0.11
**TFBUT** ^**b**^	0.08	0.26
**Fluorescein staining**	−0.06	0.41
**Schirmer I test**	−0.04	0.54

^a^OSDI, Ocular Surface Disease Index.

^b^TFBUT, Tear film break-up time.

Statistical method: Spearman’s correlation coefficient (rho).

Fluorescein staining had the highest specificity (0.97), however its sensitivity was the lowest (0.05). With OSDI cutoff score of more than 12; the AUC was 0.558 which was the highest value compared to those found for the other tests. With this cutoff the positive likelihood ratio and PPV values were also the highest (1.78, and 0.4 respectively). The specificity value was also considerably high (0.85), behind that shown by the Rose Bengal staining (cut-off ≥ 3, specificity = 0.90) (Table 5).

**Table 5:** **Diagnostic performance of all index tests**

	**Cut- off value**	**Sens** ^**a**^ **(%)**	**Spec** ^**b**^ **(%)**	**AUC** ^c**^ **(95% Confidence Interval)**	**LR+** ^**d**^	**PPV** ^**e**^ **(%)**
**OSDI**	>12 score	25.93	85.42	0.558 (0.464-0.651)	1.78	0.4
**Fluorescein staining**	≥3 score	5.17	96.64	0.509 (0.421-0.597)	1.54	0.37
**Rose Bengal staining**	≥3 score	13.79	90.60	0.522 (0.433-0.611)	1.47	0.36
**Schirmer I test**	≤10 mm	34.48	67.57	0.507 (0.415-0.598)	1.06	0.29
**TFBUT**	≤10 secs	46.55	61.49	0.535 (0.443-0.627)	1.21	0.32

^a^Sens: Sensitivity; ^b^Spec: Specificity, ^c^AUC: Area under the curve; ^d^LR+: Likelihood ratio; ^e^PPV+: Positive predictive value.

Statistical method: Diagnostic accuracy tests.

**No significant p-value in AUC was detected.

**Figure 1:**
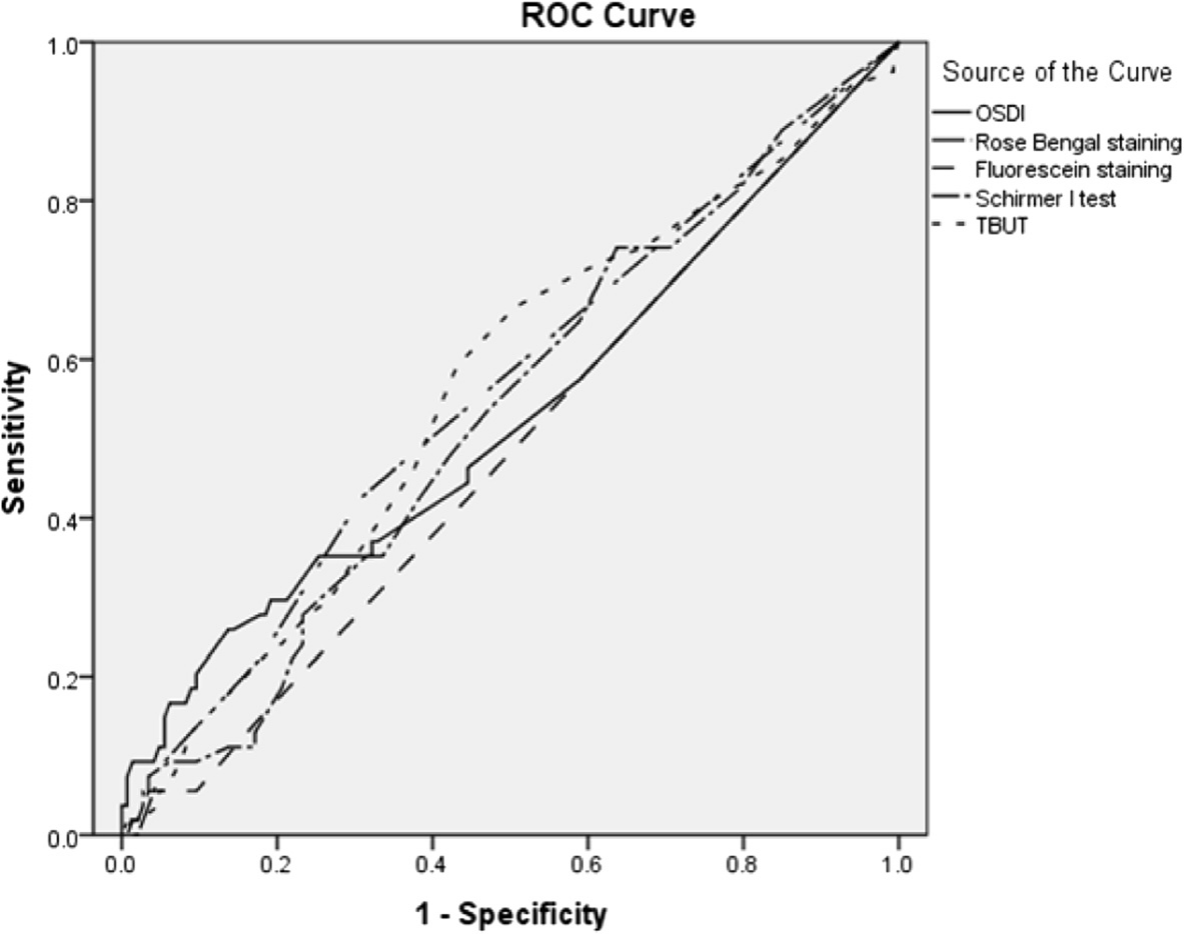
The ROC curve obtained for common diagnostic tests in dry eye, using tear osmolarity as the gold standard.

We also tried to determine the cutoffs for all index tests with an optimal sensitivity and specificity in diabetic patients using ROC curve analysis in Figure 1, but we could not define any optimal cutoff value.

## Discussion

As far as we searched the literature, this study was the first to compare the diagnostic performance of the tear osmolarity test to the other diagnostic tests used in clinical practice for detection of dry eye disease in people with type 2 diabetes mellitus. Our results revealed no significant correlation between tear osmolarity and the other diagnostic tests. OSDI had the highest AUC value with high LR+, PPV and specificity. However, it could not be suggested as a screening test due to its low sensitivity.

Dry eye disease is one of the most prevalent eye problems causing discomfort, deterioration in visual quality, and increased risk of infection [29]. Hosotani et al. [30], observed decreased tear production in diabetic patients. Goebbels M [31] suggested that amount of the reflex tearing is lower in the diabetics which can be due to diminished corneal and conjunctival sensations or may be due to neuropathy involving the lacrimal glands.

The lack of concordance between signs and symptoms in DED is a problem in the diagnosis of the disease. So, we need a reliable test or a group of tests that could be able to diagnose DED [9].

The current practical methods for diagnosing of DED such as corneal and conjunctival staining, Schirmer testing, and TFBUT are time-consuming and uncomfortable. Meanwhile, they could not predict symptomatic outcomes [32].

A new and reliable technology, tear osmolarity measurement can now is considered as a test suitable to be performed in clinical setting. The diagnostic accuracy of tear osmolarity test was found to be higher than the other tests [9].

Tear osmolarity, at a cutoff of more than 308 mOsms/L, achieved a 90.7% rate of proper diagnosis regarding severe dry eye patients in comparison with the other cutoff values (>314 mOsms/L; 86.7% and >311 mOsms/L; 89.3%), and also at the mentioned cutoff, a 73.2% rate of proper diagnosis was achieved regarding mild to moderate stage of dry eye. So, tear osmolarity is the most useful single objective test among the most commonly used tests to differentiate those with mild or moderate dry eye from those with sever disease. A cutoff threshold of more than 308 mOsms/L was found to be the most sensitive in differentiating normal from mild to moderate subjects [5].

In a comparison case- control study of diagnostic tests in keratoconjunctivitis sicca with a small sample size; tear osmolarity determination had 90% sensitivity and 95% specificity; while the Schirmer test yielded 25% sensitivity and 90% specificity [33].

In another study, tear osmolarity was found to have a 72.8% sensitivity and 92% specificity at a cutoff value of 312 mOsms/L, while corneal staining showed 54% sensitivity and 89.3% specificity, conjunctival staining 60.3% sensitivity and 90.7% specificity, TFBUT (cutoff value <10 secs) 84.4% sensitivity and 45.3% specificity, and Schirmer (cutoff value <18 mm) 79.5% sensitivity and 50.7% specificity [5].

In contrast with our findings; a diagnostic study in patients with Sjogern’s syndrome revealed that the diagnostic usefulness of Schirmer’s I test was inferior to that of TFBUT. The sensitivity and specificity were 80% and 53% respectively at a cutoff value of 5 mm in comparison with 88% and 35%, at a cutoff value of 10 mm [34].

The Schirmer test is a useful screening method for diagnosing lachrymal hyposecretion, but not for determination of the tear production threshold. Its sensitivity is between 10% and 30% [35,36]. In diabetes patients, the Schirmer test exhibits lower value than normal values [37,38]. In a series by Dogru [39], 22.7% of the eyes impregnated the strip less than 5.5 mm. In Gupta [25] and Ozdemir [40] series 9 and 34 percent of the eyes had Schirmer I value lower than 5 millimeters respectively.

The results of another study designed in six subgroups (Sjogern’s syndrome, graft-versus-host disease, Graves orbitopathy, facial palsy, diabetes mellitus without proliferative retinopathy and glaucoma who chronically received topical drugs preserved with benzalkonium chloride) associated with dry eye disease were as follows: The most sensitive test was OSDI while the least accurate was lissamine green staining . The The best combination of tests to achieve the highest combined sensitivity (100%, C.I 95% 97.5–100), specificity (95%, C.I. 95% 75.1–99.9) and accuracy (99.3 C.I. 95% 96–99.9) for DED diagnosis was OSDI/TBUT/Schirmer test. The values (% and CI of 95%.) of sensitivity for the DED tests in diabetic patients for all tests were as follows: OSDI = 69.2 (38.6–90.9), Osmolarity = 78.2 (49.2–100), TBUT = 46.1 (19.2–74.8), Schirmer test = 54.5 (23.4–83.2), and fluorescein staining = 21.4 (4.67–50.1) [41].

Besides bias due to population characteristics, the wide ranges in tear osmolarity results could be attributed to technical characteristics of the instruments and to tear collection methods, which introduce a significant and uncontrolled variable in the measurement of tear osmolarity. Trying to collect the tear volumes needed for measurements (sample size of 0.5 to 1.0 μl or larger) often results in reflex tearing, which dilutes samples and invalidates the results. Moreover, many patients with dry eye may not be evaluated due to the impossibility to properly collection of enough tear samples [9].

The strengths of this study is being the first one conducted in people with type 2 diabetes and including adequate sample size to produce reliable conclusion and influenced the degree of generalizability of the results.

A common criticism for estimating sensitivity, specificity, and prevalence without a gold standard is that, without a gold standard, it is difficult to conceptualize sensitivity and specificity [42]. But here we used gold standard to lessen the problem of conceptualizing the truth.

Potential limitations of the study include the fact that we did not consider social and environmental context of the study population; race/ethnicity or season. It is not yet known the extent to which these variables affect the distribution of osmolarity in normal persons or dry eye disease patients.

Also we are aware that studies of diagnostic accuracy are not the only type of studies to evaluate diagnostic tests.

## Conclusions

In conclusion, available common diagnostic tests for evaluation of dry eye disease underestimate the problem in people with type 2 diabetes. Moreover, they could not properly discriminate normal condition from tear hyperosmolarity and the severity of dry eye disease. So, direct measurement of the tear osmolarity could be considered as a suitable test for detection of dry eye disease in people with type 2 diabetes.

### Statement of human rights

The study was approved by the local ethical committee of Tehran University of Medical Sciences [ethical code: 39661, 31/10/2011] and conducted in accordance with the ethical principles of the Declaration of Helsinki.

### Statement of informed consent

Written informed consent was obtained from all participants for being included in the study.

## Abbreviations

DED: Dry eye disease
OGLDs: Oral glucose lowering drugs
OSDI: Ocular surface disease index
PRK: Photorefractive keratectomy
STARD: Standards for reporting of diagnostic accuracy
TFBUT: Tear film break-up time
